# Priority setting for Canadian Take-Home Naloxone best practice guideline development: an adapted online Delphi method

**DOI:** 10.1186/s12954-022-00650-4

**Published:** 2022-07-02

**Authors:** Max Ferguson, Andrea Medley, Katherine Rittenbach, Thomas D. Brothers, Carol Strike, Justin Ng, Pamela Leece, Tara Elton-Marshall, Farihah Ali, Diane L. Lorenzetti, Jane A. Buxton

**Affiliations:** 1 BC Centre for Disease Control, Vancouver, BC Canada; 2 Bloomberg School of Public Health, Johns Hopkins University, Baltimore, Maryland USA; 3 Alberta Health Services (AHS), Edmonton, AB Canada; 4 University of Calgary, Calgary, AB Canada; 5 University of Alberta, Edmonton, AB Canada; 6 Department of Medicine, Dalhousie University, Halifax, NS Canada; 7 UCL Collaborative Centre for Inclusion Health, University College London, London, UK; 8 Public Health Ontario (PHO), Toronto, ON Canada; 9 Dalla Lana School of Public Health, University of Toronto, Toronto, ON Canada; 10 Department of Family and Community Medicine, University of Toronto, Toronto, ON Canada; 11 School of Epidemiology and Public Health, University of Ottawa, Ottawa, ON Canada; 12 Centre for Addiction and Mental Health, Institute of Mental Health Policy Research, Toronto, ON Canada; 13 School of Population and Public Health, University of British Columbia, Vancouver, BC Canada

**Keywords:** Delphi, Naloxone, Harm reduction

## Abstract

**Background:**

Take-Home Naloxone (THN) is a core intervention aimed at addressing the toxic illicit opioid drug supply crisis. Although THN programs are available in all provinces and territories throughout Canada, there are currently no standardized guidelines for THN programs. The Delphi method is a tool for consensus building often used in policy development that allows for engagement of stakeholders.

**Methods:**

We used an adapted anonymous online Delphi method to elicit priorities for a Canadian guideline on THN as a means of facilitating meaningful stakeholder engagement. A guideline development group generated a series of key questions that were then brought to a 15-member voting panel. The voting panel was comprised of people with lived and living experience of substance use, academics specializing in harm reduction, and clinicians and public health professionals from across Canada. Two rounds of voting were undertaken to score questions on importance for inclusion in the guideline.

**Results:**

Nine questions that were identified as most important include what equipment should be in THN kits, whether there are important differences between intramuscular and intranasal naloxone administration, how stigma impacts access to distribution programs, how effective THN programs are at saving lives, what distribution models are most effective and equitable, storage considerations for naloxone in a community setting, the role of CPR and rescue breathing in overdose response, client preference of naloxone distribution program type, and what aftercare should be provided for people who respond to overdoses.

**Conclusions:**

The Delphi method is an equitable consensus building process that generated priorities to guide guideline development.

**Supplementary Information:**

The online version contains supplementary material available at 10.1186/s12954-022-00650-4.

## Background

A total of 26,690 people died from opioid toxicity between January 2016 and September 2021 in Canada due to the toxic drug supply [1]. Between January and September, 2021, 82% of accidental opioid overdose deaths involved a non-pharmaceutical opioid in Canada with fentanyl being involved in 86% of deaths [1].

Naloxone is a µ-opioid receptor antagonist that reverses opioid overdose and aids in preventing death with minimal adverse effects [2, 3]. In Canada, publicly-funded provincial and territorial Take-Home Naloxone (THN) programs were launched between 2012 and 2017 to provide people who use drugs and their families and friends with naloxone and training on naloxone administration [4, 5]. The efficacy of THN programs has been demonstrated globally through studies in Canada, Germany, the UK, and the USA [6–8]. In British Columbia (BC), modeling studies estimated that a total of 298 deaths were prevented by the BC THN programme between January 2012 and October 2016, and 1580 death events were prevented by the BC THN programme between April 2016 and December 2017 [2, 9].

The Canadian THN Guideline development project was funded through the Canadian Research Initiative in Substance Misuse Emerging Health Threat Implementation Science program in response to escalating mortality associated with opioid toxicity. THN programs are widely recognized as an important response to the to toxic drug supply yet there are discrepancies within THN programs in Canada, such as whether intranasal THN kits are available and the number of ampoules/vials of naloxone included in THN kits [4].

Guidelines are based on current evidence and used to optimize consistent service provision and administration [10]. There are currently no Canadian guidelines on THNs. The World Health Organization and American Society of Addiction Medicine released guidelines that discussed THN, and the European Monitoring Centre for Drugs and Drug Addiction published a report on current evidence on THN in 2014, 2015, 2016, respectively [11–13]. Further, Tsuyuki et al. released Canadian guidelines for naloxone prescribing by pharmacists [14].

While past THN guidelines offer valuable information, we identified a gap in guidelines developed specifically for community THN distribution programs, which have different informational needs and challenges compared to professional groups. The Canadian harm reduction context may differ from other countries as there is regional variation regarding the scope and quantity of harm reduction policies [15], available harm reduction services [16], and THN distribution programs [4] within Canada. Additionally, harm reduction science continues to develop. In this study, we aim to integrate new evidence and engage stakeholders from across the country to generate new guideline recommendations [10]. A guideline with updated evidence review will help address the current surge in deaths due to illicit opioid toxicity in the Canadian context [1].

### The Delphi method

The Delphi method is a consensus building approach used to inform policy decisions [17, 18]. Through gathering diverse expert perspectives, the Delphi method leverages a range of direct knowledge and experience while stimulating discussion between members. This leads to stronger, context-relevant decision-making; an essential outcome for guideline development where the end product must be applicable over multiple jurisdictions [18]. Anonymous decisions are elicited, individual views are aggregated and presented back to the group and the process is repeated a recommended two times; this allows for an exchange of ideas while ensuring anonymity and equitable space to voice alternate views [18].

Guideline development groups should be multi-disciplinary and represent diverse views, involving a variety of methodological experts and clinicians, as well as any population affected by the guidelines [19]. The Delphi method facilitates equitable participation by ensuring that everyone’s input and perspectives will be included, and reducing the potential for uneven power dynamics or dominant personalities to unduly influence the process [17]. This is important in clinical and academic projects related to harm reduction, where the voices of people with lived or living experience of substance use (PWLLE), while necessary for guideline validity, are often absent or disrespected [20]. Previous studies have used the Delphi approach in the field of harm reduction [21, 22].

## Methods

### Study aim

The aim of the adapted online Delphi method was to investigate which research questions stakeholders believed should be prioritized in a Canadian THN guideline.

### Study setting

All meetings and data collection were conducted online in order to get perspectives of stakeholders from across Canada and to respect physical distancing measures during the COVID-19 pandemic. Author MF facilitated all subcommittee meetings with support from author JN. Authors AM, CS, KR, TDR sat on the Guideline Steering Committee.

### Guideline development group formation

Guideline Development Group recruitment was an iterative process aiming for pan-Canadian membership and diverse perspectives. Four Guideline Development Group subcommittees participated in the Delphi method.

Recruitment occurred between November 2020 and January 2021. The study principal investigators and the affiliated Canadian Research Initiative in Substance Misuse (CRISM) working group members nominated members for the Guideline Development Group. These initial members nominated other potential stakeholders to expand and diversify recruitment using a snowball sampling approach.

PWLLE were explicitly invited via the snowball sampling process. PWLLE who were connected or unconnected to advocacy, research, or frontline harm reduction organizations were eligible for participation. We recruited from existing communities of PWLLE such as the Canadian Association of People who Use Drugs, the People with Lived and Living Expertise of Drug Use National Working Group, and Professionals for Ethical Engagement of Peers, from our professional networks, and sought feedback from identified PWLLE to ensure diversity of experiences and cross-country representation.

We invited participants from all subcommittees to introduce themselves in whatever manner made them feel comfortable, including lived and living experience, but did not explicitly ask all group members whether they identify as people who use drugs. We recruited sufficient self-identified PWLLE to ensure representation in all subcommittees. PWLLE in the Guideline Development Group who were not being compensated for participation through their workplace were provided $25/hr honoraria [23].

Effort was made to find representation across Canadian provinces and territories through the local and national networks of existing members. Indigenous, Black, and People of Color and 2S/LGBTQ group members were explicitly invited in the snowball sampling process.

Subcommittees of the Guideline Development Group included:

1. The Guideline Steering Committee (composed of eight members) steered discussions, encouraged constructive debate, summarized main points and key decisions, and gave oversight to the guideline development process.
2. The Affected Community Committee (composed of seven members) provided guidance and recommendations based on the values and preferences of people with lived experience related to naloxone distribution and use in opioid overdose.
3. The Clinical Expert Committee (composed of 13 members) provided guidance and recommendations on the use of naloxone in opioid overdose from a clinical perspective.
4. The Guideline Development Panel (composed of 15 members) voted on the key questions to be addressed by the guideline  as part of a Delphi method. Members of the Guideline Steering Committee, Affected Community Committee, and Clinical Expert Committee were invited to participate in voting as part of the Guideline Development Panel.

Author AM, a member of the Guideline Steering Committee, introduced meeting facilitation strategies drawing on experiences facilitating with community groups and varied stakeholders on harm reduction topics in British Columbia [24]. Meetings began with territory acknowledgements, and facilitation techniques such as introductions, icebreakers and co-creating group agreements were introduced into the Guideline Development Group meetings to foster safer space for group members to share perspectives and to address potential power dynamics [24]. This collaborative process acknowledged and honored the varied experiences, cultures, and strengths of committee members and provided the foundation for safer engagement [24] and promoted a more relational process of knowledge generation [25]. We invited participants to participate in whatever manner made them most comfortable whether that included simply speaking up, using “raise hand” function in ZOOM video conferencing software, or typing in ZOOM chat.

### Key question development

We identified initial key questions based on a recent scoping review of the literature on the use of naloxone for reversal of opioid overdose in a community setting [26]. Themes identified in the scoping review included general opioid overdose response strategies, naloxone administration methods, naloxone distribution program strategy and implementation, cost-effectiveness of program implementation, and acceptability of naloxone distribution programs [26].

Three subcommittees (Guideline Steering Committee, Affected Community Committee, and Clinical Expertise Committee) reviewed these initial questions and suggested new questions before the Guideline Development Panel voted on the importance of each question. The Guideline Development Group discussed whether to include questions on cost-effectiveness due to concern about the potentially stigmatizing effect of comparing lives lost to public health programming cost. However, some Guideline Development Group members described challenges getting funding for services in the absence of evidence on cost-effectiveness. Questions on cost remained in the voting process but were flagged for careful consideration for presentation of evidence.

The Guideline Steering Committee, Affected Community Committee, and Clinical Expert Committee recommended that plain language questions rather than academic research questions or statements be developed to guide the process [10]. The Guideline Development Group found it easier to evaluate the importance of the questions if they were in plain language format.

### Delphi method

A total of 15 Guideline Development Panel members participated in voting on which key questions should be addressed in the guideline. The Guideline Development Group was composed of people that had overlapping lived or living/clinical/academic experience. A table with descriptions of the panel members’ professional and advocacy roles is included as a Additional file 1.

**Fig. 1:**
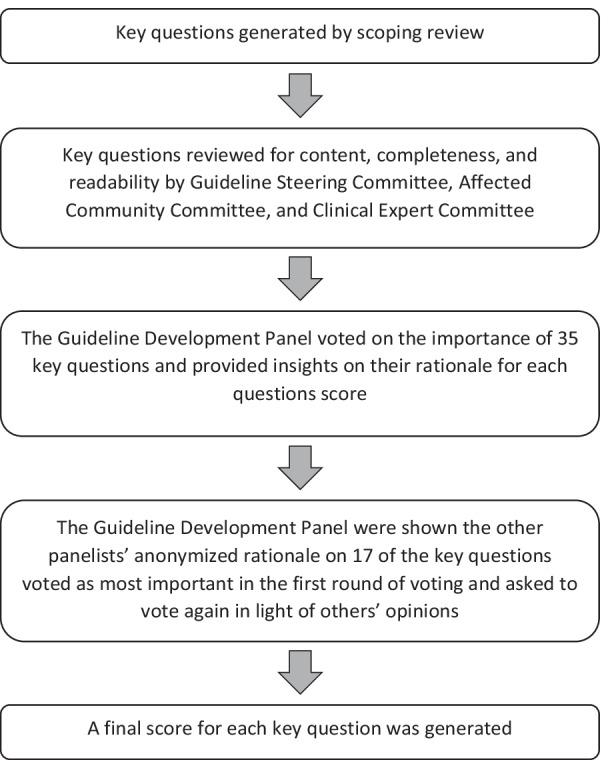
Delphi method

Methodology for the Delphi method was drawn from McMillan et al. [17]. Data collection tools were developed and piloted by research team members not involved in tool development and administered online via REDCap (Research Electronic Data Capture) software. The Guideline Development Panel was presented with 35 key questions and asked: “Do you agree that this question is important to discuss in a best practice guideline?” (see Fig. 1). Response options were presented in a 5-point Likert scale (strongly agree/agree/neutral/disagree/strongly disagree where strongly agree = 1 and strongly disagree = 5) and respondents were then asked: “Can you please explain how you came to the rating for the previous question?” Consensus was operationalized a priori as a mean score below 2.0 [17]. Key questions which scored 2.0 or above were excluded from the second round of voting. In the second round, panel members were asked to consider the reasons their peers agreed/disagreed with the importance of the remaining key questions and rate these questions once more. The mean scores for each question were then calculated, using R, version 4.0.5 [27]. See Fig. 1 for an overview of Delphi method.

The method was adapted from early literature on the Delphi by moving data collection online to accommodate for the national scope of the project and to avoid limitations related to the ability to meet in-person during the COVID-19 pandemic [17].

All Guideline Development Panel members participated in the first round of voting (15/15) while 80% of members (12/15) participated in the second round of voting.

The research team used the CREDES tool to inform reporting of the Delphi method in this manuscript [28].

## Results

A total of 17 of the 35 original research questions scored below 2.0 in the first round of voting and so were retained for the second round. Nine of the 17 key questions were rated below 2.0 in the second round of voting and qualified for inclusion in the THN guideline. The mean scores for both rounds are presented in Table 1. The questions with the lowest score in the second round of voting (and therefore the highest perceived importance) were on what should be in naloxone kits and the effectiveness of different methods of naloxone administration.

**Table 1:** Key questions scored independently by Guideline Development Panel

Key questions	Mean score round 1^a^	Mean score round 2^a^
**General opioid overdose response strategy**		
What is the effectiveness of Take-Home Naloxone programs?	1.6*	1.6*
Are there different rates of mortality and morbidity for persons experiencing overdose in community setting associated with:		
Rescue breathing in addition to naloxone administration Conventional CPR including rescue breathing in addition to naloxone administration Compression-only CPR in addition to naloxone administration Naloxone administration alone?	1.4*	1.8*
What should be in naloxone kits?	1.4*	1.4*
After naloxone administration, how long should people be observed to ensure the reversal was effective? What happens if people are or are not transported to the hospital?	1.6*	2.0
What aftercare should be provided for people who respond to overdose?	1.6*	1.9*
**Naloxone administration methods**		
Are health outcomes different when different dosages of naloxone are used?	2.3	–
What is the effectiveness of different methods of naloxone administration, including dosage, repeat doses and titration (gradual increase in dosage), length of time before onset of action (how long until the medication starts working), and serum half-life (how long it takes for half of the dose to be eliminated from the bloodstream) of naloxone at achieving reversal of overdose?	1.5*	1.4*
Are there storage issues that impact effectiveness of naloxone in a community setting?	1.8*	1.7*
What are important safety considerations, including those for adverse reactions (unexpected or unwanted effects) and opioid withdrawal symptoms?	2.1	–
Is there a difference in preference for administration methods for different populations, e.g., between people who use drugs vs family or friends of people who use drugs, people who inject drugs vs people who inhale?	2.6	–
**Naloxone distribution program strategy and implementation**		
What are the crucial factors and different possible structures for naloxone distribution programs?	1.8*	2.2
What specific program objectives and what measurable outcomes are being used to inform success (reordering of kits, population coverage/uptake, satisfaction of staff/public, mortality, consensus)?	2.0	–
What types of programs (Take-Home Naloxone (THN), Facility Overdose Response Box (FORB), others) exist or are needed?	1.8*	2.4
What distribution model gets the most kits to people who use them? How does this differ among different populations (including people who are incarcerated, rural populations, Indigenous populations)?	1.5*	1.6*
What eligibility criteria for PWUD^b^ impact kit distribution?	2.8	–
What is the effect of overdose response training and education strategies, on primary outcomes (number of deaths due to opioid overdose avoided) and intermediate outcomes (number of kits dispensed, number of kits reported used)?	1.7*	2.2
What are the risks and benefits of provincial listings of naloxone as a Schedule II vs unscheduled drug, and what effect, if any, does it have on primary outcomes (number of deaths due to opioid overdose avoided) and intermediate outcomes (number of kits dispensed, number of kits reported used)?	1.9*	2.6
How does stigma impact how PWUD^b^ and families and friends of PWUD^b^ access distribution programs?	1.6*	1.5*
What are the barriers and facilitators of access to distribution programs?	1.6*	2.0
What resources are required for the effective distribution and access to naloxone?	1.7*	2.0
What policies are required for the effective distribution and access to naloxone?	1.7*	2.1
Do people prescribed opioids for pain have access to THN? Should all people prescribed opioids be routinely offered naloxone kits or should it be based on risk factors?	2.2	–
How should distribution models differ in rural vs urban settings?	2.0	–
**Cost-effectiveness of program implementation**		
What is the cost-effectiveness of the intervention?	2.0	–
What evidence exists of social and economic benefits of program implementation and overdose prevention and what are economic evaluations of costs and cost–benefit ratios from health care and societal perspectives?	2.2	–
What is the relative cost-effectiveness between administration methods (nasal vs intramuscular)?	2.6	–
What is the cost-effectiveness of funding of naloxone kits, including individual doses, assembly of kits, pharmacy dispensing fees, and training fees?	2.5	–
What is the cost for implementation and running the naloxone distribution program?	2.4	–
What are the costs for data collection and program monitoring, as well as researcher/agency capacity to evaluate distribution programs?	2.9	–
**Acceptability of naloxone distribution program**		
What kind of naloxone distribution program do people impacted want? Is this impacted by PWUD demographics?	1.9*	1.8*
What is the demand for and acceptability of naloxone distribution programs among people who use drugs, people who may witness an overdose, health professionals in community settings, the general public, government, and policy authorities?	2.4	–
How is the need for naloxone distribution programs communicated to the general public and how effective is that communication?	2.5	–
What are the legal or political benefits or risks of beginning or expanding programs?	3.4	–
How to make naloxone distribution easy, accessible and palatable for families of PWUD?	2.0	–
Does communication about naloxone kits/disclosure of possession/training of family impact health outcomes?	2.3	–

*Scores under 2.0 which the guideline development panel identified as important to prioritize in the THN guideline

^a^Where 1 = strongly agree and 5 = strongly disagree that the question is important

^b^People who use drugs

## Discussion

The Delphi method was conducted to identify what questions community stakeholders thought important to prioritize in a Canadian guideline on THN. The following key questions were identified as important to address: What equipment should be in THN kits, whether there are important differences between intramuscular and intranasal naloxone administration, how stigma impacts access to distribution programs, how effective are THN programs at saving lives, what distribution models are most effective and equitable, storage considerations for naloxone in a community setting, the role of CPR and rescue breathing in overdose response, client preference of naloxone distribution program type, and what aftercare should be provided for people who assist with overdoses.

Group heterogeneity among those making decisions can lead to more thorough consideration of all relevant aspects of a topic including applicability; by including a diversity of stakeholders, we were able to thoroughly weigh the pros and cons of key questions from different perspectives [18]. The Delphi method is especially important for ensuring meaningful inclusion of PWLLE within clinical and academic contexts and has been applied elsewhere in harm reduction research [21, 22]. PWLLE who provide harm reduction services including overdose response are uniquely knowledgeable about harm reduction practices and services, engaging communities of people who use drugs (PWUD), and creating comfortable and safe service environments [29, 30]. As we have seen in a recent analysis of BC Take-Home Naloxone kit distribution data, PWUD are the group most likely to receive a kit because their previous kit was used to reverse an overdose; thus PWUD are key stakeholders, both as the people who will benefit from THN programs and policies and who save lives using THN [31, 32]. Other committee members offered valuable insights from clinical (with representation from harm reduction, nursing, pharmacy, and medicine), public health, and academic perspectives from different provinces and territories within Canada.

Prior Delphi studies in harm reduction report including people from advocacy groups [21] or advocates and people with lived and living experience [33]. The expertise of PWUD is invaluable in creating meaningful and impactful service delivery and they should have a central voice in programs and policies that affect them [20].

We found the online Delphi method to be especially beneficial in the context of a pan-Canadian project occurring during dual public health emergencies of drug toxicity and the COVID-19 pandemic. Public health and community health organizations reported feeling increasingly burdened trying to respond to the pandemic while concurrently seeing increases in overdose-related mortality [34, 35]. The online Delphi method eliminated travel or time-consuming videoconferencing. Engagement of the committee members was flexible and safe during the COVID-19 pandemic and did not incur travel and meeting costs. Additionally, it did not require participants to leave their home community which can be difficult for those who use substances.

### Next steps

The Delphi method is the first step in community engagement for a Canadian THN guideline and will inform the next stage of the guideline development project. Three of the highly ranked key questions will be translated to research questions for systematic reviews and the evidence will be evaluated using the GRADE approach [36]. Evidence from the systematic reviews will be used to inform recommendations on THN in Canada. These recommendations will be brought back to the Guideline Development Group for input based on their content expertise and knowledge of the Canadian harm reduction context.

### Limitations

Despite targeted recruitment, Newfoundland and Labrador, the Northwest Territories, Nunavut, and Prince Edward Island were not represented in the Guideline Development Group. Some of the nominees from these provinces and territories reported a higher than usual workload due to the COVID-19 pandemic as a barrier to participation. While two of the three territories were not represented, perspectives from the Yukon were included. Conclusions drawn through this Delphi method may not be generalizable to the provinces and territories not represented.

A key limitation is reliance on online meetings. While use of online meetings and data collection allowed us to engage people from across Canada, it could present barriers to engagement especially for those with limited access to technology or limited technological knowledge including PWLLE. Additionally, our project relied on participants’ comfort with sharing ideas. While data collection was conducted using survey software, other meetings occurred over ZOOM video conferencing software. Prior qualitative research shows that while participants may feel able to express ideas during online focus groups, they also felt less engaged with other participants and less able to have a conversation that flowed compared to face-to-face discussions [37]. We observed that using ice breakers and spending time on introductions helped group members share ideas more readily.

The research team drafted the guideline questions using Population/Problem, Intervention/Exposure, Comparison, Outcome/Findings (PICO) language, then reworded them into accessible language for the Delphi method. However, this created uncertainty with the research team around the interpretation of some of the prioritized questions. The team was unable to ascertain how individuals interpreted the common language questions, which led to challenges in creating the final PICO questions used in the literature searches in the absence of more extensive pilot testing. Capturing comments on the questions or asking the voters to explain their understanding of the questions may have ensured that voters all had similar interpretations. Additionally, key questions included multiple outcomes, but the research team needed to narrow down questions for feasibility of executing the systematic reviews that will generate evidence for the THN guidelines.

## Conclusion

The Delphi method allowed Guideline Development Group members from across disciplines, life experiences, and geographical space to share insights and help establish priorities for a Canadian guideline. A guideline on Take-Home Naloxone informed by key stakeholders will provide evidence to support consistent, effective, and equitable service delivery across Canada. The evidence on the nine questions identified through this Delphi method study will be explored through systematic review.

## Supplementary Information

**Additional file 1**.** Table 1**. Guideline Development Panel Breakdown.

## Data Availability

All data generated or analyzed during this study are included in this published article (and its Additional file 1).

## Abbreviations

PWLLE: People with lived and living experience of substance use
PWUD: People who use drugs
PICO: Population/problem, intervention/exposure, comparison, outcome/findings
THN: Take-Home Naloxone
