# Supplementary material for: Priority setting for Canadian Take-Home Naloxone best practice guideline development: an adapted online Delphi method
Source: Harm Reduct J. 2022 Jul 2;19:71. doi: 10.1186/s12954-022-00650-4 (PMC9250272; doi:10.1186/s12954-022-00650-4)
Supplement: Supplementary file 1 — Additional file 1. Table 1. Guideline Development Panel Breakdown. [file 12954_2022_650_MOESM1_ESM.docx]

Table 1: Guideline Development Panel Breakdown

| **Panel member** | **Role Description** |
| --- | --- |
| 1 | Public health professional |
| 2 | Clinician and academic with expertise in harm reduction |
| 3 | Person with lived and living experience, frontline worker, harm reduction advocate |
| 4 | Public health professional |
| 5 | Clinician and academic with expertise in harm reduction |
| 6 | Executive director of a harm reduction Non Governmental Organization |
| 7 | Clinician and academic with expertise in harm reduction |
| 8 | Academic with expertise in harm reduction |
| 9 | Public health professional and academic with expertise in harm reduction |
| 10 | Academic with expertise in harm reduction |
| 11 | Person with lived and living experience, harm reduction advocate, and academic with expertise in harm reduction |
| 12 | Clinician and academic with expertise in harm reduction |
| 13 | Clinician and academic with expertise in harm reduction |
| 14 | Clinician and academic with expertise in harm reduction |
| 15 | Clinician and academic with expertise in harm reduction |
